# Computational engineering of low bandgap copolymers

**DOI:** 10.3389/fchem.2013.00035

**Published:** 2013-12-13

**Authors:** Michael Wykes, Begoña Milián-Medina, Johannes Gierschner

**Affiliations:** Madrid Institute for Advanced Studies, IMDEA NanoscienceMadrid, Spain

**Keywords:** conjugated materials, donor-acceptor copolymers, low bandgap polymers, quantum-chemistry, density functional theory, polymer extrapolation, optical bandgaps

## Abstract

We present a conceptual approach to low bandgap copolymers, in which we clarify the physical parameters which control the optical bandgap, develop a fundamental understanding of bandgap tuning, unify the terminology, and outline the minimum requirements for accurate prediction of polymer bandgaps from those of finite length oligomers via extrapolation. We then test the predictive power of several popular hybrid and long-range corrected (LC) DFT functionals when applied to this task by careful comparison to experimental studies of homo- and co-oligomer series. These tests identify offset-corrected M06HF, with 100% HF exchange, as a useful alternative to the poor performance of tested hybrid and LC functionals with lower fractions of HF exchange (B3LYP, CAM-B3LYP, optimally-tuned LC-BLYP, BHLYP), which all significantly overestimate changes in bandgap as a function of system size.

## Introduction

Low bandgap (co)polymers have attracted much attention for use in polymer-based bulk heterojunction (BHJ) organic solar cells (OSCs) to efficiently harvest the near infrared portion of the solar spectrum. The possibility to combine donor (D) and acceptor (A) units in a DA copolymer structure (Roncali, 1997), and to introduce functional side chains with electron donating/withdrawing moieties and/or sterical demands, which mainly control the polymer morphology, has opened sheer endless possibilities to effectively tune the optical bandgap (Roncali, 1997; Ajayaghosh, 2003; Bundgaard and Krebs, 2007; Kroon et al., 2008; Chen and Cao, 2009; Cheng et al., 2009; Son et al., 2011; Zhou et al., 2012), but also to achieve a broad absorption spectrum (Beaujuge et al., 2010), balanced electronic levels (Li, 2012; Takimiya et al., 2013), improved processability, and controlled packing (Chen et al., 2009).

However, the optoelectronic properties and processes in OSCs are a complex matter; the polymers' excited state features (energies and oscillator strengths of singlet and triplet states), the absolute position of the highest (lowest) (un)occupied molecular orbitals, HOMO (LUMO), interchain packing, and interfacing with the BHJ acceptor material (usually fullerene variants), sensitively influence not only the photogeneration of excitons, but also exciton transport, charge generation, recombination, transport and extraction at the electrodes; for a recent review, see (Coughlin et al., 2013). Facing these challenges, quantum chemical (QC) calculations have emerged as an indispensable tool to understand properties and processes in OSCs (Brédas et al., 2009; Risko et al., 2011), but also to prescreen polymers prior to synthesis. However, the relevant length- and time-scales of the various polymer properties and optoelectronic processes span several orders of magnitude, and despite steadily increasing computer speeds, only the shortest of these length- and time-scales can be treated with the most accurate of today's methods. Thus, the rather modest task of calculating optical transitions of single (co)polymer chains taxes even cost-effective methods, previously based on semi-empirical configuration interaction singles theory (CIS), but now almost exclusively based on (time-dependent) density functional theory, (TD)DFT.

Nevertheless, despite providing significantly higher accuracy than semi-empirical methods in many cases, DFT and its TD extension suffer from several pitfalls (Dreuw and Head-Gordon, 2004; Cohen et al., 2012). One such pitfall is that currently popular and widely used DFT functionals such as B3LYP typically provide higher accuracy for small molecules than large ones and as such they are unable to correctly predict changes in properties as a function of system size. In particular, they provide an incorrect evolution in bond-length alternation (BLA) (Jacquemin et al., 2006; Sancho-García and Pérez-Jiménez, 2007; Körzdörfer et al., 2012), ionization potentials (Körzdörfer et al., 2011), and HOMO-LUMO band gaps and electronic transition energies as a function of system size (Gierschner et al., 2007; Körzdörfer et al., 2011). The latter failure limits the utility of TD-DFT methods in the accurate prediction of the optical properties of conjugated polymers by extrapolation of oligomeric transition energies. Gierschner et al. (2007) showed clearly for several classes of conjugated polymers that B3LYP overestimates the decrease in transition energy as the chain length increases, mainly due to the overestimation of MO delocalization (Milián-Medina and Gierschner, 2012a), resulting in significantly underestimated extrapolated polymer values.

While the experts' discussions are occupied by a forest of new functionals adapted to solve the pitfalls of (TD)DFT for specific applications, B3LYP has become the workhorse for non-experts often without consideration of its limitations. At the same time, combinatorial approaches are screening millions of small oligomer building blocks around the clock (Hachmann et al., 2011; Kanal et al., 2013). Both parties seem in some way to hamper the legitimate quest for both reliable prediction of the optoelectronic properties at the polymer limit and a proper understanding of the underlying physics. It also does not help that mutual understanding between experiment and theory is often lacking.

This article aims to develop such understanding in the specific case of low bandgap (co)polymers. For both experiment and theory, we adopt the oligomer approach, where the (optical) bandgaps of oligomers are plotted against the reciprocal chain length *n* (Meier et al., 1997; Müllen and Wegner, 1998; Meier, 2005). Only by studying evolution with *n* can the pitfalls of the QC methods be properly detected. In fact, in literature, the extreme care required when comparing calculated and measured optical bandgaps is rarely taken. Gas phase calculations on an oligomer are often directly compared to experimental polymer values without consideration of environmental effects and hence little can be learned about the reason for the success or failure of different QC methods.

There are essentially only two relevant physical parameters which control the chainlength evolution and the polymer bandgap, which can be extracted by a Kuhn-fitting procedure (Gierschner et al., 2007). In light of this parameter set we will discuss concepts for low bandgap materials, unify the terminology by comparing the formally divided donor-acceptor (DA) and benzoid-quinoid (BQ) strategies, demonstrate the need for a proper QC description of the 2-parameter set and shortly discuss MO localization phenomena. In the last section, selected DFT-based QC methodologies will be systematically tested by a careful comparison with experimental values for oligothiophenes and for low bandgap co-oligomers, allowing us to suggest a viable approach to polymer bandgap prediction.

## Computational details

### Methodology

Ground state optimizations as well as frontier orbital calculations (energy and topology) were performed using DFT with various functionals, including B3LYP with 25% HF exchange (Becke, 1993a,b; Stephens et al., 1994), BHLYP [as implemented in Gaussian09, similar but not identical to Becke (1993b)] with 50% HF exchange, M06HF (Zhao and Truhlar, 2006) with 100% HF exchange, the long-range corrected (LC) CAM-B3LYP (Yanai et al., 2004), and finally a LC optimally tuned (OT)-LC-BLYP for which the range parameter was chosen to tune both the HOMO and LUMO and hence the bandgap for each oligomer. This was achieved by minimization of the function

(1) $$\begin{array}{l} J^{2}(\mu )={\left[\varepsilon_{HOMO}^{\mu }(N)+IP^{\mu }(N)\right]}^{2}+[\varepsilon_{HOMO}^{\mu }(N+1)\\ {\;+\;IP^{\mu }(N+1)]}^{2} \end{array}$$

where μ is the range parameter, ε_*HOMO*_ is the HOMO energy, IP is the vertical ΔSCF ionization potential, and *N* is the number of electrons, (Foster and Wong, 2012) using a home-built code incorporating an automatic minimization routine. The code was tested and reproduced the optimized range-separation parameters obtained in (Foster and Wong, 2012) for five systems. Optimized OT-LC-BLYP geometries were obtained by alternating between successive geometry and J^2^(μ) optimizations to self-consistency. The 6-31G^**^ basis set was used throughout. Vertical absorption transition energies E_vert_ were then calculated using all the above functionals coupled to a TD-DFT scheme. Semiempirical E_vert_ were calculated by the ZINDO/S method, i.e., intermediate neglect of differential overlap method as parameterized by Zerner et. al. for spectroscopic applications (Zerner, 1994) coupled to an single configuration interaction (SCI) scheme taking into account all π/π^*^ type orbitals. All (TD)DFT calculations were done in vacuum within the Gaussian09 program package (Frisch et al., 2009). Orbital pictures were drawn with MOLEKEL 4.3 (Flukiger et al., 2000–2002).

### Geometries

All calculations were performed on hydrogen-terminated oligomers. Ground state equilibrium structures of dimeric (co-)oligomers were optimized to determine the starting structures for the longer oligomers. For the TD-DFT studies, geometries were optimized with various functionals described above in order to establish to what extent structural differences, in particular BLA and inter-ring torsion angles, affect excitation energies and their chain-length evolution. In general, higher BLA and larger torsion angles will shorten the conjugation length and lead to larger bandgaps. Full optimization of geometries within both planar and non-planar symmetry groups allowed the impact of differences in predicted BLA and torsions to be distinguished. Accordingly, thiophene oligomers (*n*T, *n* = 2–6) and thienopyrazine-alkyl-thiophene co-oligomers (*n*TTP, *n* = 1–5) were optimized within the planar groups C_2v_ and C_2h_ (odd and even *n*, respectively)and the non-planar groups C_s_/C_2_. Diketopyrrolopyrrole thiophenes (*n*TDPP, *n* = 1–3) were optimized in the planar and non-planar groups C_2h_ and C_2_, respectively. Oligophenylenevinylenes (*n*PV, *n* = 2–5) were optimized in the planar and non-planar groups C_2h_ and C_i_, respectively.

For the ZINDO/S bandgaps geometries were optimized at the B3LYP/6-311G^*^ level. Here, (alkoxy-)thiophene oligomers (*n*T, *n*ROT) were kept coplanar as shown earlier (Milián Medina et al., 2008); also thiophene methines (*n*TM) were found to be planar systems and were calculated at the maximum possible symmetry (C_2h_, C_2v_, C_s_). Benzene-based systems, benzothiadiazole (*n*BT) and its co-oligomers (F8BT) were found to be far from planarity (inter-ring torsional angles of 33 and 35°, respectively) (Huang et al., 2011); no symmetry restrictions were imposed here. Isothionaphthenes (ITN) and it's co-oligomers with thiophene were also found to be non-planar.

## Key parameters of bandgap engineering

**Figure 1:**
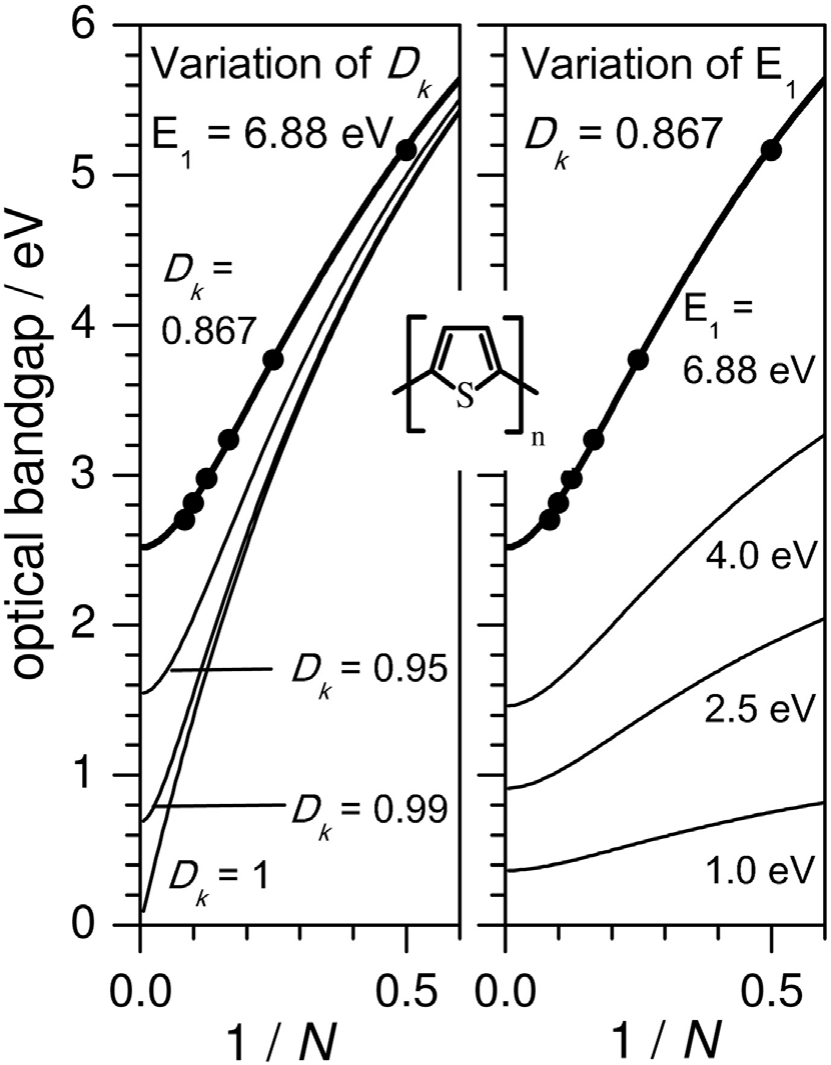
**Optical bandgaps as a function of 1/*N* with *N* as the number of double bonds on the shortest conjugation pathway between the terminal carbons**. Solid circles: Experimental values (*in vacuo*) for oligothiophenes *n*T (where *n* = 2N) (Gierschner et al., 2007). Solid line: Fit according to the Kuhn equation (Figure 2) with *E*_1_ = 6.88, *D*_*k*_ = 0.867. Variation of the Kuhn parameters *D*_*k*_ (left) and *E*_1_ (right).

**Figure 2:**
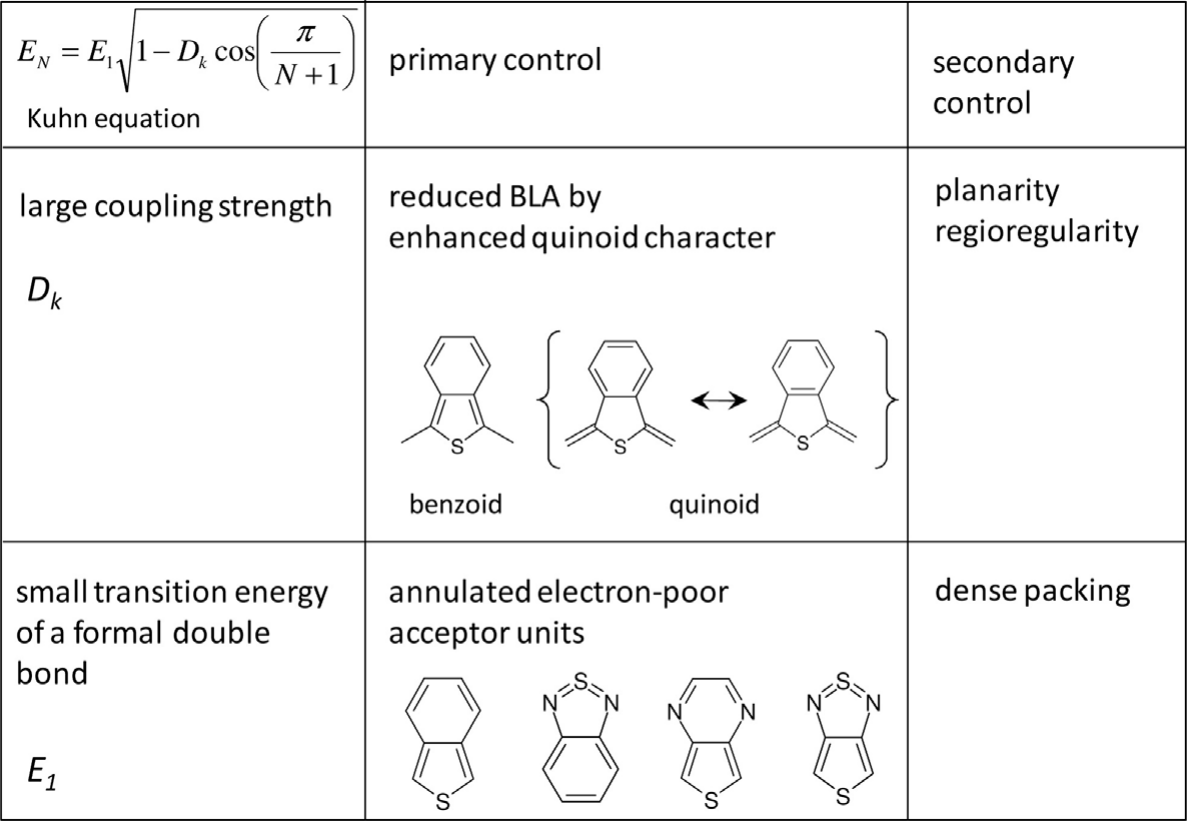
**Parameters of the Kuhn equation and their primary and secondary control in conjugated (co)polymers**.

Figure 1 shows the evolution of the experimental optical bandgap *E* (adiabatic transition energies) of unsubstituted oligothiophenes (*n*T) where *n* is the number of the repetition units (in this case thiophene rings), where the optically allowed singlet transition from the ground to first excited state (S_0_→S_1_) of *n*Ts [and the majority of the conjugated (co)polymers] is sufficiently described by the HOMO→LUMO transition. Bandgaps in *vacuo* were obtained from the intersections of absorption and emission spectra utilizing different solvents and extrapolation to *vacuo* as described elsewhere (Gierschner et al., 2002, 2003, 2007). This procedure allows for a precise comparison with QC calculations of adiabatic (as well as vertical) transition energies, which are conventionally performed in *vacuo*. Plotting *E_N_* against 1/*N*, *N* being the number of double bonds along the shortest conjugated pathway between the terminal carbon atoms (for *n*T, *N* = 2 · *n*), a fairly linear relation for small oligomers (*N* ≤ 12) is obtained (see Figure 1). This however saturates for long oligomers above the effective conjugation chain length (which can be graphically determined) (Gierschner et al., 2007), reaching the polymer limit at E_8_ = 2.5 eV for *n*T (in *vacuo*). It has been shown (Gierschner et al., 2007), that the chain length evolution can be reasonably described by a simple 2-parameter model according to Kuhn (1948). This model is based on a linear coupling of harmonic oscillators (double bonds) responsible for the electronic transition (Figure 2), where *E*_1_ is the transition energy of a formal double bond (*N* = 1) and *D_k_* is a relative force constant (*D*_*k*_ = 2 · *k_s_/k_d_*), measuring how strongly the double bonds (isolated oscillators of force constant *k_d_*) are coupled by the single bonds through the force constant *k_s_*.

The saturation for *E*(*N*→∞) in Kuhn's coupled oscillator model has its quantum-chemical equivalent in the localization of the frontier MOs in the center of the molecule with increasing *N*. This confines the change in BLA upon S_0_→S_1_ excitation (i.e., from a benzoid pattern with a large BLA to a more delocalized pattern with a small BLA) to a finite number of units at the center of the molecule (Milián-Medina and Gierschner, 2012a). In fact, DFT-calculated HOMO-LUMO gaps also show a Kuhn-like evolution (Milián Medina et al., 2007), also for very large *n* (Zade et al., 2011), as recently reviewed (Torras et al., 2012). Within the Kuhn model, *D_k_* is implicitly related to the change in BLA upon S_0_→S_1_ excitation; smaller changes in BLA are associated with values of *D_k_* closer to unity, and hence smaller bandgaps. Thus, in order to promote smaller bandgaps, a smaller ground state BLA is indeed required as stated frequently in literature (Kertesz et al., 2005); starting from a ground state which already has a low BLA decreases the change in BLA upon excitation, and results in a *D_k_* closer to unity.

It should be noted that for certain classes of materials, empirical fitting functions, e.g., the 3-parameter exponential fit introduced in Meier et al. (1997) give somewhat better fits to the experimental values (Karsten et al., 2008). However, we want to stress that in contrast to the exponential fit, the Kuhn model allows for a physical interpretation of the 1/*N* evolution and thus molecular design rules, *vide infra*. The sometimes unsatisfactory Kuhn fit reflects to the simplicity of the model, which assumes a constant *D_k_* with increasing *n*. Although the latter is true for a large variety of materials (Gierschner et al., 2007), there are important exceptions all arising from “end effects.” Firstly, monomeric species (*n* = 1), can usually not be fitted with the same parameters as longer oligomers due to pronounced resonance stabilization in the monomer, which is weakened for longer chains. Other exceptions are oligomers with strong terminal donor and/or acceptor substituents, whose impact on the π-electron system significantly decreases with chainlength (Meier, 2005; Gierschner et al., 2007), and thus do not allow for a simple homogenous description. A third exception arises from systems, in which the BLA changes strongly between the rings at the end of the chain and those at the center, e.g., in terminal tetracyano-methylene-substituted oligothiophenes (TC*n*T), which introduce a quinoid structure in the end groups (Ponce Ortiz et al., 2007). All these cases require a modification of the Kuhn equation, with e.g., an additional exponential term (Gierschner et al., 2007).

**Figure 3:**
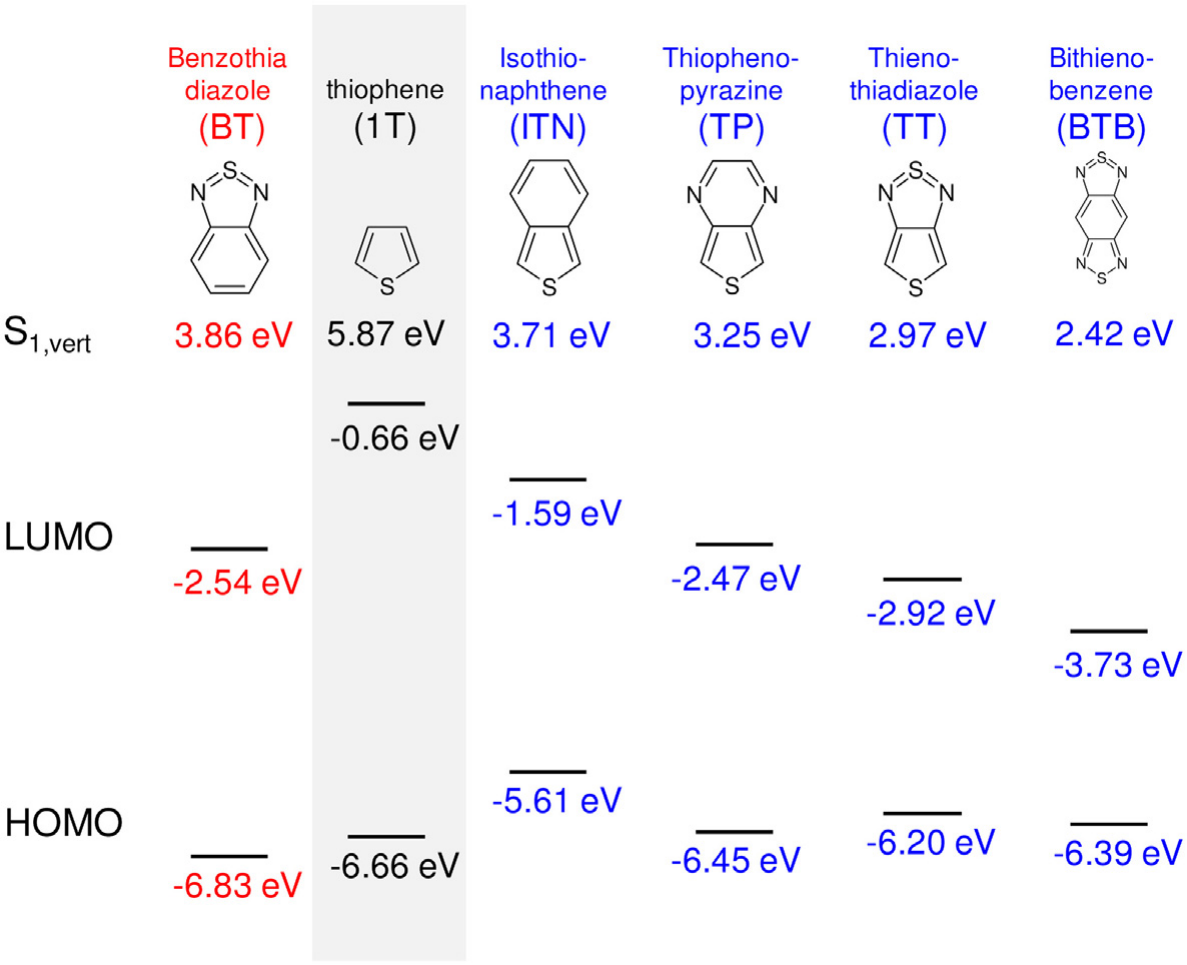
Examples of small bandgap monomers, with calculated HOMO and LUMO levels as well as E_vert_ obtained at the (TD-)DFT B3LYP/6-311G^*^ level of theory.

**Figure 4:**
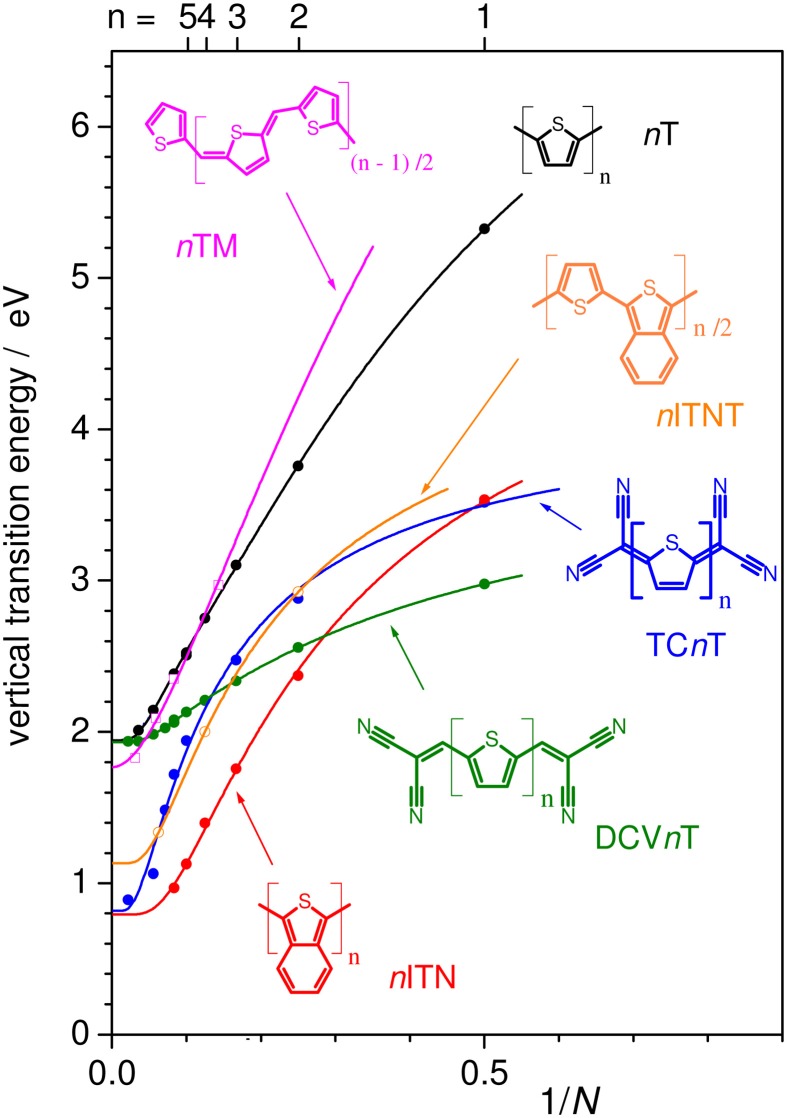
E_*vert*_ of different conjugated oligomers as a function of 1/N as calculated at the ZINDO/S level on DFT (B3LYP/6-311G^*^) optimized geometries. Solid lines for *n*T and *n*TM are fits according to the Kuhn equation (Figure 2); for the DCV*n*T mono-exponential fits, and for *n*ITN and TC*n*T biexponential fits were used.

For the vast majority of conjugated materials with *n* ≥ 2 however the Kuhn model performs reasonably well (Gierschner et al., 2007). This immediately provides two simple strategies for achieving lower bandgaps, which are summarized in Figure 2:

- an *increase of D_k_* (while keeping *E*_1_ constant) leads to an increase of the slope and diminishes the curvature at the polymer limit (Figure 1), thus increasing the effective conjugation length for *D_k_* < 1 and yielding a zero bandgap for the limiting case of *D*_*k*_ = 1. This corresponds to a decreased BLA by increasing the quinoid character of the conjugated chain (*vide infra)*, as predicted early on for poly-isothionapthene (*P*ITN; Figure 3) (Kürti and Surján, 1990), or for the previously mentioned TC*n*T series, which both show a substantial reduction of the lowest optical transition relative to the *n*T series as predicted by relatively simple semi-empirical QC calculations, see Figure 4.
  Besides this primary effect to manipulate *D_k_*, the planarity of the molecular backbone constitutes a secondary effect, which can have a considerable impact on the slope. This can be seen e.g., in the strongly sterically hindered oligophenylenes (Milián-Medina et al., 2011); here the introduction of a torsional twist leads to a significant decrease of *D_k_*. This effect should be distinguished however from only thermally induced non-planarity, which blue-shifts the absorption maximum (which, to a first approximation, corresponds to E_vert_), but not the optical bandgap (which corresponds to the adiabatic transition energy) as previously demonstrated (Gierschner et al., 2002, 2003). For side-chain substituted systems, regio-regularity is crucial to promote planarity of the chain as has been shown for polyhexylthiophene (*P*3HT) (Gierschner et al., 2007).
- a *decrease of E_1_* (and thus of the excitation energy of the repetition unit *E_RU_*) leads to smaller bandgaps at the polymer limit; however the slopes of the chainlength evolution get significantly smaller as long as *D_k_* is kept constant, see Figure 1. To reduce *E_RU_*, electron withdrawing moieties are used, e.g., by replacing benzene-based RUs by thiophene, or better, by annulated rings which reduce *E_RU_* mainly due to the stabilization of the LUMO (Figure 3), and/or using electron-donating substitution patterns. For instance, electron-donating side-chain substituents in all rings lead to somewhat lower bandgaps also at the polymer limit (compare e.g., *P*3HT vs. *P*T with a gain of ca. −0.1 eV due to the inductive +I effect of the alkyl groups) (Gierschner et al., 2007). On the other hand, terminal substitution with strong D/A groups can have a very large effect on small oligomers in generating small bandgaps (Meier, 2005), however this effect will vanish at the polymer limit (Meier, 2005; Gierschner et al., 2007). This is also true for the recently discussed dicyano- (Pina et al., 2006) or dicyanovinyl-thiophene (DCV*n*T) series (Pappenfus et al., 2003; Schulze et al., 2006), see Figure 4. A secondary effect in this category is the promotion of dense packing in the solid state by minimizing the disorder, which significantly lowers the effective optical bandgap due to enhancement of the anisotropic polarizability of the material via closely packed parallel chains (Egelhaaf et al., 2002), whereas H/J-aggregation play only a very minor role in polymers (Gierschner et al., 2009).
  In summary, low bandgaps with E_∞_ ≤ 1 eV are only expected if narrow bandgap repetition units are combined with a small BLA. An example for a homopolymer fulfilling these conditions is poly-thiophenopyrazine (*P*TP; Figure 3), with a small optical bandgap of ca. 1.2 eV in solution, (1.0 eV in films) (Wen et al., 2008). The low bandgap of *P*TP is further promoted by planarization due to attractive sulfur-nitrogen interactions (Özen et al., 2007), evidenced by well-structured vibronic side bands of the absorption spectrum (Milián Medina et al., 2008; Wen et al., 2008). Appropriate side chains will further promote dense packing and thus lowered bandgaps; however this has to be balanced with the solubility requirements for polymer processing.

## Conceptual approaches to low bandgap polymers

### Benzoid-quinoid and donor-acceptor copolymers

Although homopolymers like *P*ITN might reach very low bandgaps, the application of such materials is limited, both due to the rather limited possibilities for electronic tuning (e.g., of the absolute MO energies) and processability through side chain functionalization. Thus, copolymers emerged as an alternative, combining different conjugated repetition units and substitution patterns to provide better processability and a large variation in optoelectronic properties and packing motifs. Copolymers can be formally divided into BQ and DA copolymers (Kertesz et al., 2005; Zhou et al., 2012). Herein, BQ copolymers are formed by insertion of quinoid repetition units into a benzoid backbone; (Kertesz et al., 2005), where (partial) quinoid character in the repetition units is introduced through resonance-stabilized annulated rings (e.g., the benzene ring in the ITN unit in Figure 2), whose aromaticity can be additionally enhanced by electron-donating substituents. It should be noted however that e.g., poly(thiophene methine), *P*TM (Chen and Jenekhe, 1995), see Figure 4, although it connects B and Q units, behaves very similar to the *n*T series (Figure 4). This is due to the fact that the BLA path of both subunits is not broken, i.e., the alternation is pursued throughout the chain, since B and Q units do not compete for the same bonds. Thus, *P*TM might be rather called a pseudo-BQ copolymer.

**Figure 5:**
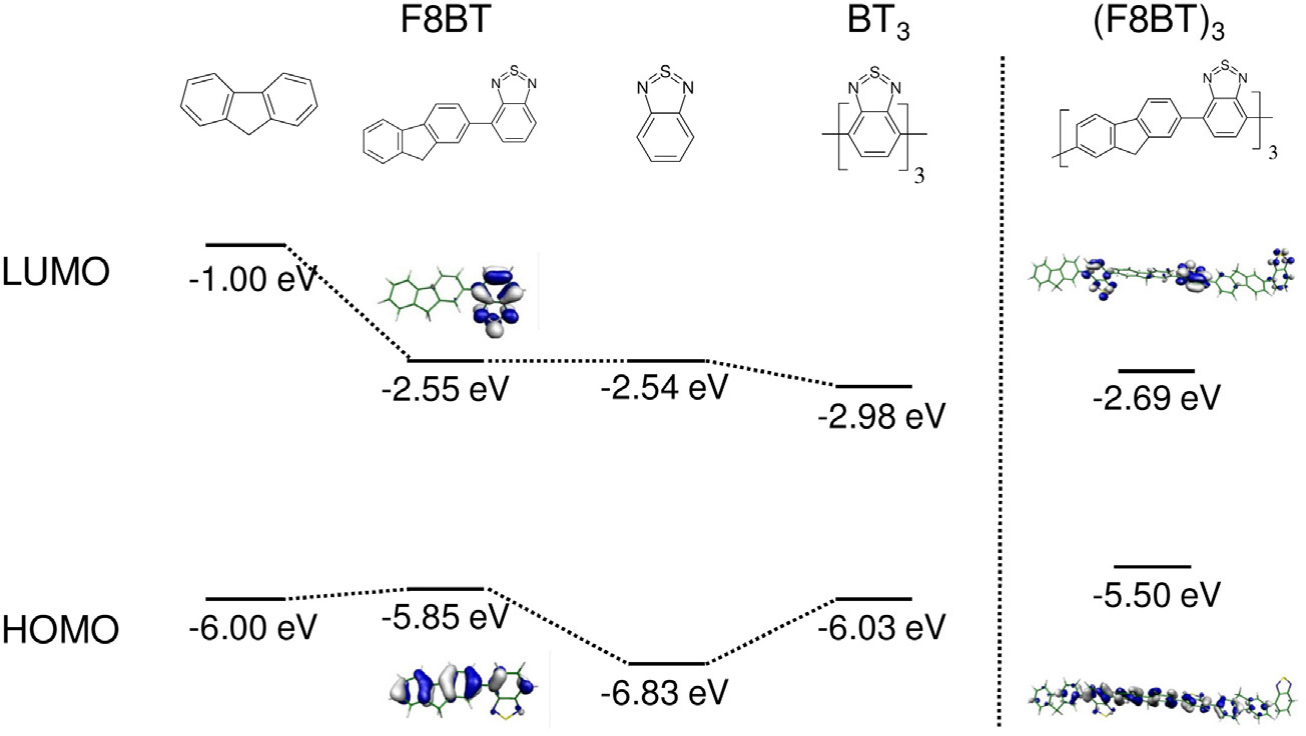
**Left:** Orbital correlation diagram (B3LYP/6-311G^*^, no symmetry restrictions) for the (F8BT) and BT_3_ from their oligomer fragments. **Right:** frontier orbitals of (F8BT)_3_.

In DA copolymers, decreased energy of the repetition unit is accomplished by appropriate DA pairs with low lying HOMO and LUMO levels of A compared with those of D, as shown for D = fluorene (F8) and A = benzothiadiazole (BT) in Figure 5. Consequently, the LUMO energy of DA is similar to that of A, and the HOMO to D, thus yielding a small bandgap for DA (Havinga et al., 1993; Cornil et al., 2003; Karsten et al., 2009). To realize narrow bandgaps, annulated electron-withdrawing moieties as acceptor units are used (Figure 3), which usually belong to the same family as the quinoid moieties, so that in practice, a distinction between BQ and DA copolymers is not required; in fact, as we will see later all copolymer pairings have an impact in both *E_1_* and *D_k_*.

### Bandgap tuning via the copolymer concept

Although bandgaps of DA pairs are indeed smaller compared to those of single D and A units, this is not true at the polymer limit as one could suspect in a first approximation (Havinga et al., 1993). Instead, acceptor-only A_∞_ homopolymers exhibit lower bandgaps than (DA)_∞_ copolymers (Karsten et al., 2009). In fact, it is not the relative energy of the (isolated) D and A frontier MOs which is relevant in determining the optical bandgap of the copolymer (Salzner and Köse, 2002), but *E*_1_ and *D_k_*, as we can conveniently see for F8BT (Figure 5). Following the idea of the last section, the bandgap of the DA (F8BT) repetition unit has to be compared to an (A)_n_ oligomer with the same number of double bonds *N*, since this governs the chainlength evolution. In the present example this is (BT)_3_, which has in fact a smaller bandgap than F8BT (Figure 5) due to the larger coupling *D_k_* of the BT oligomers compared to the coupling between F8 and BT. Thus, the F8BT copolymer bandgap is expected to be found between that of polyfluorene and the (hypothetical) BT-polymer. The same principle holds for other copolymers, e.g., composed of (alkyl-substituted) thiophene (T) and thienopyrazine (TP) units, where *D_k_* of poly-TP (*P*TP) is considerably smaller than that of polythiophene (*P*T). Indeed, for the copolymer *P*[T_2_TP_1_], both *D_k_* and the absorption maximum (and thus the bandgap) lie between the corresponding homo-polymers (Karsten et al., 2009). However, the bandgap for *P*[T_2_TP_1_] is smaller than might be expected from its stoichiometry [i.e., *E*(*P*[T_2_TP_1_]) < 2/3·*E*(*P*T) + 1/3·*E*(*P*TP)], which is due to a substantial quinoid character of TP introduced into the copolymer chain (Karsten et al., 2009), *vide supra*. Further detailed discussions of the variation in bandgaps as a function of D/A stoichiometry can be found in the quantum-chemical studies of Gil-bernal et al. (2010) and Hung et al. (2013).

Hence, to reach small bandgaps via the DA concept, small bandgaps of the A moiety are decisive, assisted by quinoid contributions as discussed above. It should be noted that the quinoid character introduced in the copolymer chain might significantly vary with the chemical composition of the D/A moieties, and thus change *D_k_*. Hence, prescreening copolymer MO properties through correlation with simple DA pair calculations as is now frequently done (Blouin et al., 2008; Hachmann et al., 2011; Kanal et al., 2013), might not result in appropriate selections for fully-optimized polymers (*vide infra*). On the other hand, screening of copolymers via periodic calculations (Longo et al., 2012; Bérubé et al., 2013) encounters the same problem from the other side, relying on a correct evolution of the electronic and optical properties with chain length. Thus, a reliable prediction of both *E_1_* and *D_k_* by QC methods will be crucial, *vide infra*.

### Frontier orbital localization

Electronic level tuning by the DA concept might imply orbital localizations of the frontier MOs within one of the moieties (Dutta et al., 2009; Milián-Medina and Gierschner, 2012b). The main factor for MO localization is the energy offset between the frontier orbitals of the HOMOs (LUMOs) of D and A segments; other factors include orbital symmetries and conjugation breaks by sterical demands (Schmidtke et al., 2007). F8BT might once more serve as an example, see Figure 5. Here the HOMO is formed by both the F8 and the BT unit, whereas the LUMO is localized on the BT unit (Cornil et al., 2003); thus the HOMO→LUMO transition shows partial intermolecular charge transfer (ICT) character. However, MO topologies might significantly change when going from a simple DA pair to the polymer limit, and so the chainlength evolution has to be carefully studied (Karsten et al., 2008; Dutta et al., 2009). For longer F8BT oligomers, the LUMO is indeed localized on the acceptor moieties, but homogeneously distributed on all BT units within the conjugation length, rather than on a single BT unit (Figure 5). This topology differs significantly from a report on a phenylene-ethynylene based copolymer with a highly localized HOMO (and delocalized LUMO) (Dutta et al., 2009). There, the localization was spatially limited to one single unit of the polymer chain and thus produced a low-lying ICT state of small oscillator strength, which gave rise to a large Stokes shift between emission and main absorption band.

The question in which way MO localization influences the electronic properties of the copolymer in an OSC BHJ blend is an intriguing one. As the charge-carriers in the copolymer phase are holes, LUMO localization will probably not be a problem, while (partial) HOMO localization might be detrimental. In any case, the polar structure induced by the DA topology could be even helpful for the charge separation at the BHJ interface (Carsten et al., 2011). Moreover, localization resulting in spatial separation of HOMO and LUMO promotes smaller singlet-triplet energy gaps (Milián-Medina and Gierschner, 2012b), which might be of some importance since the low lying triplets seems to play a crucial role in the mechanism of charge recombination in OSCs (Veldman et al., 2009).

## Prediction of (co)polymer bandgaps

The conceptual tools developed above allow us to assess the performance of different QC methods to properly describe the chainlength evolution of (co)oligomers, and to identify their pitfalls. These failures of standard DFT are known, being due to the approximate form of the exchange potential resulting in spurious self-interaction, and in particular, its incorrect distance-dependence. The admixture of exact Hartree-Fock (HF) exchange into the functional (in the so called hybrid functionals), typically improves the accuracy (Milián-Medina and Gierschner, 2012a). LC functionals aim to provide a more balanced description of exchange by varying the fraction of exact HF exchange as a function of distance, and have been applied with some success to long-range charge transfer states for which both popular pure and hybrid functionals typically fail (Dreuw and Head-Gordon, 2004). We will thus especially appraise whether recently developed LC functionals coupled to a TD scheme provide an accurate description of the evolution of optical bandgaps as a function of oligomer length, and hence whether they can offer the predictive power necessary for use as a screening tool in the design of novel conjugated (co)polymers for OSC applications.

**Figure 6:**
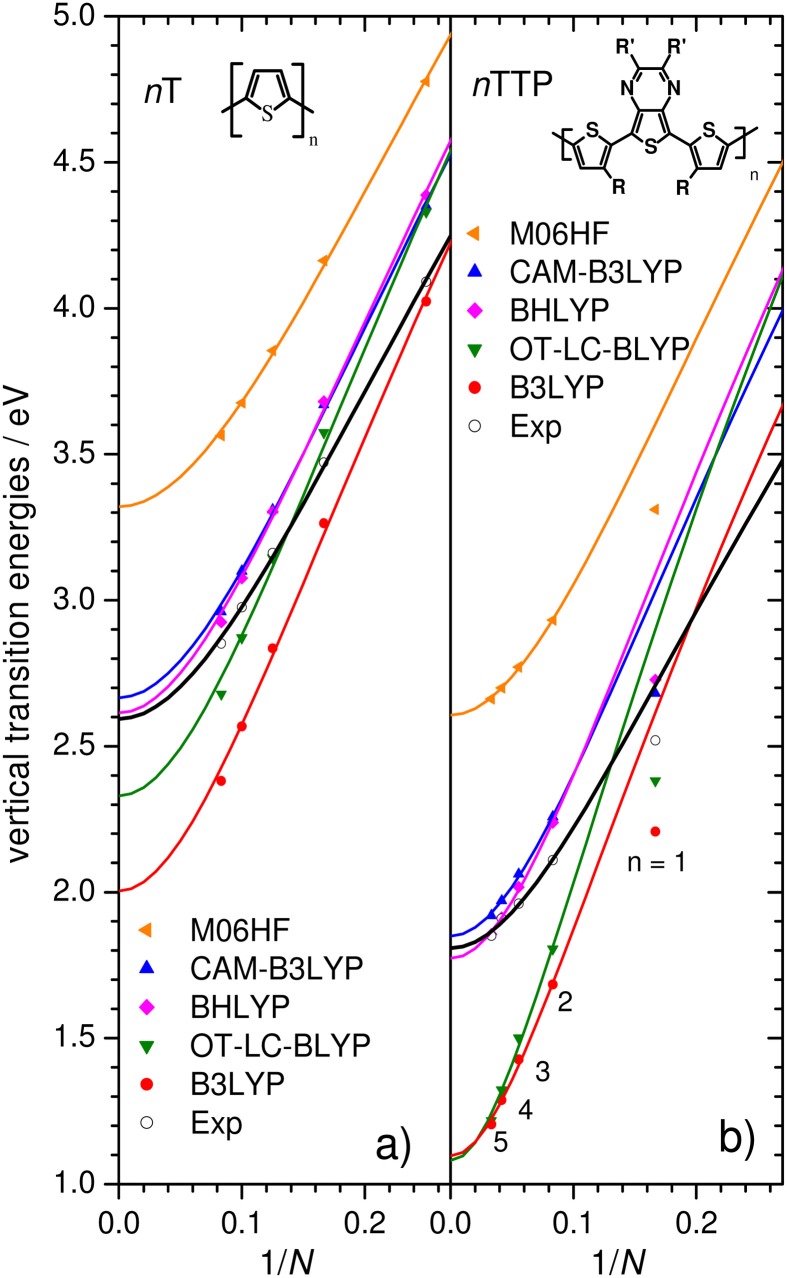
**E_vert_ of *n*Ts (A) and *n*TTPs (B) computed using a range of DFT functionals for both geometry optimization (planar C_2v_/C_2h_) and transition energies (solid symbols) and Kuhn fits (lines)**. Experimental results (open symbols; *n*Ts: in DCM, *n*TTP: in toluene) are shown for comparison.

In order to assess the reliability of QC methods, we will perform a careful comparison with experimental oligomer evolution. As a test case for a homopolymer we will use E_vert_ of the *n*T series (Gierschner et al., 2003), followed by a comparison for low bandgap co-oligomers. Unfortunately, there are only a few examples of systematic experimental studies on co-oligomers in literature; notable exceptions are systems synthesized in the group of R. Janssen, i.e., the *n*TTP series (Karsten et al., 2008), see Figure 6, and diketopyrrolopyrrole thiophenes *n*TDPP (Karsten and Janssen, 2011).

Improper comparison can produce non-negligible errors which can sum up to 1 eV at the polymer limit, see Gierschner et al. (2007). This concerns the proper definition (and experimental extraction) of the optical transitions (adiabatic vs. vertical transition vs. HOMO-LUMO gap), environmental effects (solvent and solid-state shifts, temperature), and substituent effects. Here we strive for a compromise between accuracy and computational efficiency in order to develop a procedure suitable for rapid screening of (co)polymers with extended repeat units. As such, we focus on vertical absorption energies in solution, and assume that bathochromic solvent shifts and temperature effects do not vary significantly with chain-length, nor between (co-)oligomer series, and can thus be modeled as a rigid shift in transition energies. The available data on *n*Ts (*n* = 2–6) suggests that this is a reasonable approximation; vacuum to solvent shifts in E_vert_ are 0.19 ± 0.01 eV at 293 K in dichloromethane (DCM) and the 15K–293 K shift of E_vert_ in DCM is 0.16 ± 0.01 eV (Gierschner et al., 2007). Equivalent data is not available for the *n*TTP, *n*TDPP series, however this approximation seems to work quite well (*vide infra*).

Experimental Evert bandgaps of the *n*T and *n*TTP series are shown as open symbols in Figure 6 with the corresponding Kuhn fits through data points with *n* ≥ 2. The difference between the two series is striking, giving not only much lower values of *E_0_* for the *n*TTP series (see Table 1) but also stronger couplings *D_k_*, indicative of significant quinoid contributions. Concomitantly, the slope *m* of the *n*T series (obtained from a straight-line fit in the quasi-linear regime of the data points; *n* = 2 to ca. 6), is 50% higher than that of *n*TTP. This directly demonstrates that reliable predictions of polymer bandgaps via the oligomer approach cannot be obtained from calculations on monomers (*n* = 1), but requires at least the calculation of two data points (*n* = 2, 3).

**Table 1:** **Kuhn fit parameters *E_1_* (in eV) and *D_K_* extracted from the experimental and computed E_vert_ as given in Figure 6**.

**Experiment**			***n*T**						***n*TTP**					
			***E_1_***	***D_k_***	***E_∞_***	***m***	**Δ*E_off_***	**σ**	***E_1_***	***D_k_***	***E_∞_***	***m***	**Δ*E_off_***	**σ**
			**7.7**	**0.89**	**2.59**	**7.4**			**6.8**	**0.93**	**1.81**	**4.9**		
**COMPUTATIONAL METHOD**														
**TD^a^**	**GO^b^**	**S^c^**												
B3LYP	B3LYP	p	8.3	0.94	2.00	9.8	0.30	0.16	7.6	0.98	1.10	7.4	0.51	0.14
	BHLYP	p	8.4	0.93	2.19	9.6	0.15	0.15	7.7	0.97	1.33	6.9	0.32	0.11
CAM-B3LYP	CAM-B3LYP	p	8.3	0.90	2.67	8.4	−0.17	0.06	7.9	0.94	1.85	5.7	−0.11	0.04
	B3LYP	p	8.3	0.91	2.46	8.8	−0.01	0.09	8.1	0.96	1.56	6.6	0.11	0.09
OT-LC-BLYP	OT-LC-BLYP	p	8.7	0.93	2.33	9.9	−0.01	0.16	8.6	0.98	1.08	8.6	0.42	0.20
	B3LYP	p	8.5	0.93	2.29	9.6	0.05	0.15	8.4	0.98	1.18	8.0	0.38	0.17
BHLYP	BHLYP	p	8.5	0.91	2.61	8.8	−0.16	0.09	8.2	0.95	1.77	6.5	−0.08	0.09
	BHLYP	np	8.7	0.89	2.82	8.6	−0.35	0.08						
	B3LYP	p	8.4	0.93	2.19	9.6	0.15	0.15	8.3	0.97	1.46	7.3	0.17	0.13
M06HF	M06HF	p	8.6	0.85	3.32	7.3	−0.70	0.01	8.3	0.90	2.61	4.9	−0.81	0.01
	M06HF	np	8.7	0.81	3.77	6.6	−1.07	0.06						
	B3LYP	p	8.5	0.88	2.91	8.2	−0.38	0.05	8.6	0.94	2.03	6.4	−0.33	0.08
	B3LYP	np	8.8	0.88	2.99	8.4	−0.49	0.07						
	BHLYP	p	8.6	0.87	3.15	7.8	−0.58	0.02	8.5	0.92	2.37	5.6	−0.61	0.04
	BHLYP	np	8.8	0.85	3.36	7.6	−0.77	0.02	8.6	0.92	2.39	5.4	−0.63	0.03

E_∞_ is the extracted polymer value (in eV) at the polymer limit; m is the slope from a straight-line fit. ΔE_off_ is the difference of E_vert_(measured)–E_vert_(calculated) averaged over each oligomer series and σ is its standard deviation, representing an average error within a series arising from incorrect chain length evolution.

a TD, time-dependent calculation.

b GO, geometry optimization.

c S, symmetry; p, planar; np, non-planar; see geometry subsection for details.

Figure 6 also plots calculated E_vert_ using a range of DFT functionals. For clarity, results are shown only for planar geometries optimized using the same functional as used to compute the transition energies (a subset of the calculations shown in Table 1). The B3LYP functional strongly overestimates the slope for both series (Figure 6), as previously reported for various homo-oligomers (Gierschner et al., 2007; Milián-Medina and Gierschner, 2012a). OT-LC-BLYP (for which the range-separation parameter had been optimized for each oligomer) performs similarly poorly to B3LYP, significantly overestimating *D_k_* and the slope. As such, B3LYP and OT-LC-BLYP are the two worst-performing functionals we tested when it comes to chain-length evolution of optical bandgaps. The CAM-B3LYP functional gives significantly smaller *D_k_* values (Table 1), yet still strongly overestimates the slopes relative to experimental values. Thus, in contrast to what one might expect, the LC functionals tested here do not correct for the errors in popular hybrid functions which lead to significant overestimation of changes in bandgaps as a function of system size. It is important to note that while the CAM-B3LYP extrapolated polymer value *E_8_* for the *n*TTP series is close to experiment [ΔE(*n*TTP) = 0.04 eV], in light of the poor performance on the slope, this must be ascribed to a fortuitous cancellation of errors. However, these errors in *E_8_* do not always cancel, but are strongly system dependent; for the *n*T series, they result in the rather unsatisfying error ΔE(*n*T) of 0.22 eV. Thus, we cannot recommend these functionals for unbiased pre-synthesis polymer prediction.

**Figure 7:**
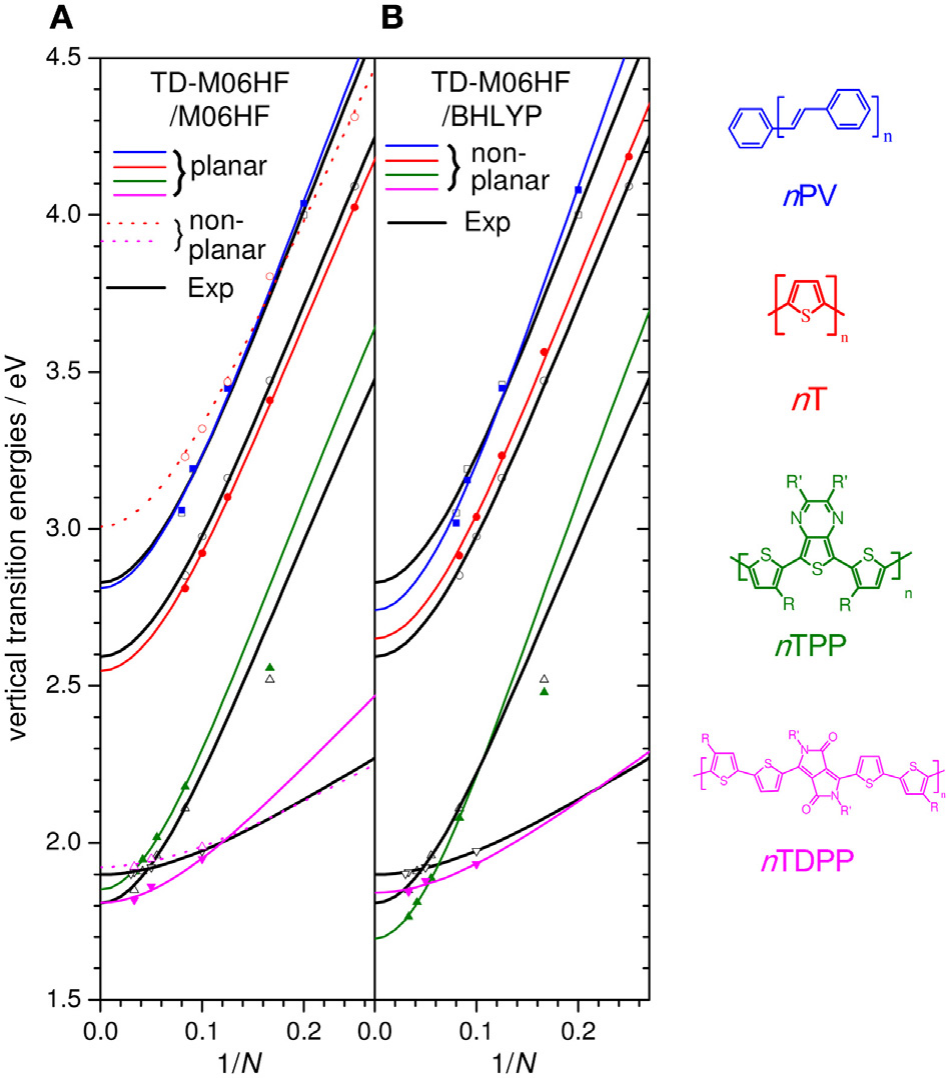
**Experimental E_vert_ (black open symbols) and offset-corrected M06HF results for the *n*PV,*n*T, *n*TTP, and *n*TDPP series (R,R′ = alkyl chains) using; (A) planar M06HF geometries (solid symbols) and non-planar M06HF geometries (colored open symbols), both with ΔE_off_ = 0.75, and (B) non-planar BHLYP geometries using ΔE_off_ = 0.70**. Lines are Kuhn fits to the data points.

As the fraction of exact HF exchange is increased from 20% in B3LYP to 50% in BHLYP, the slope decreases, but still significantly overestimates *m* compared to experiment, see Table 1. Increasing the HF exchange to 100% by using M06HF (for both the geometry and TD parts) yields slopes in remarkable agreement with experiment for both *n*T and *n*TPP, consistent with the previous finding that RCIS/HF also provides accurate slopes (Gierschner et al., 2007). Although M06HF bandgaps feature a strong hypsochromic offset relative to experiment, the shift is surprisingly similar for the *n*T (ΔE_off_ = 0.70 eV) and the *n*TTP series (0. 81 eV) despite the strong differences between the two series with respect to both *E_1_* and *D_k_*. We have further tested the M06HF performance on other homo- and co-oligomer series (oligophenylenevinylene; *n*PV, and *n*TDPP), showing similarly good results with Δ E_off_ (*n*PV) = 0.76 and Δ E_off_ (*n*TDPP) = 0.69, as depicted in Figure 7 where the experimental values are compared with the offset-corrected M06HF results with ΔE_off_ = 0.75 eV. The average and maximum absolute deviation of predicted E_∞_ from experimental value are 0.05 and 0.09 eV, suggesting that this method fulfills our criteria for accurate polymer screening (accuracy to within 0.1 eV). It should be stressed that ΔE_off_ is basis set dependent. Thus, while the value of Δ E_off_ = 0.75 ± 0.10 eV is valid for the 6-31G^**^ basis set used in Figure 7, our preliminary tests on *n*Ts using a 6-311G^**^+ basis require a value Δ E_off_ = of 0.46 ± 0.09 eV. It has been shown that the RCIS/HF method also provides correct slopes in transition energies as a function of chain length (Gierschner et al., 2002, 2003). As for TD-M06HF, RCIS/HF transition energies also overestimate the measured values. This observation was not apparent in the original reports nor in subsequent papers (Gierschner et al., 2007; Milián-Medina and Gierschner, 2012a) as the reported energies are in fact vertical emission energies E_vert,em_, and not adiabatic energies (E_00_) as stated therein (by chance, for *n*Ts, E_vert,em_ from RCIS/HF/6-311+g^*^ are almost identical to measured E_00_ gas phase values).

While the slopes for *n*T, *n*TTP, and *n*PV are in excellent agreement with experiment, the slope of *n*TDPP is significantly overestimated. This is likely due to our performing calculations on fully planar geometries, while *n*TDPP oligomers with *n* > 1 are significantly twisted due to steric hindrance where two alkyl-thiophenes connect. Indeed, using non-planar geometries results in a chain-length evolution in excellent agreement with experiment for *n*TDPs (see Figure 7, dashed magenta line). It should be noted however that the same procedure applied for the *n*T series results in an underestimation of the chain length evolution and significant increase in the hypsochromic shift relative to experiment (see Figure 7, dashed red line). This is due to the pronounced deviation from planarity; e.g., in 2T the inter-ring torsion is 156°, and similar values are found in 6T. This runs contrary to experimental evidence that while 2T is twisted, longer oligomers are essentially planar: the lack of mirror symmetry of the absorption and fluorescence spectra of 2T shows that its ground state is far from planar in agreement with *ab initio* benchmark calculations and experiments (Raos et al., 2003). But as the chain length is increased, the absorption and fluorescence spectra become increasingly symmetric, suggesting that the ground state is, like the excited state, planar in longer oligomers (Macchi et al., 2009). The results suggests that M06HF does not provide sufficiently accurate torsional potentials to correctly describe bandgap chain-length evolution in quasi-planar systems without symmetry constraints, but for such systems M06HF can provide the correct chain-length evolution if planarity is enforced.

The role of geometry was further investigated by combining different functionals for transition energy calculations and geometry optimizations and comparing results of planar and non-planar symmetries (see Table 1). TD-BHLYP using non-planar BHLYP geometries provides slightly smaller slopes than planar geometries, but still overestimates them relative to experiment, suggesting torsions alone are not responsible for the slope overestimation. For planar geometries, slopes decrease in the order TD-B3LYP/B3LYP > TD-B3LYP/BHLYP > TD-BHLYP/B3LYP > TD-BHLYP/BHLYP, suggesting that the choice of functional used in the TD part has a larger impact than the functional used for geometry optimization. Nevertheless, the geometry clearly has a strong impact; TD-M06HF calculations on planar and non-planar B3LYP, BHLYP, and M06HF *n*T geometries show a significant spread in *E_∞_*, slopes and offsets relative to experiment. Interestingly, TD-M06HF on non-planar BHLYP geometries provided quite good results for *n*Ts (almost as good as TD-M06HF on planar M06HF geometries). This combination was therefore also tested on *n*TTP, *n*PV, and *n*TDPP in the hope that it might provide a general approach to polymer extrapolation, free from assumptions about planarity. Figure 7B plots the results (with an offset of −0.70 eV). Consistent with the data in Table 1, the chain-length evolution of *n*Ts is indeed very well described, and as hoped for, the slope for *n*TDPP is significantly improved relative to the planar M06HF/M06HF calculations (Figure 7A). However, this does come at the cost of a slight loss of accuracy for *n*TTPs and *n*PVs. Nevertheless, the average and maximum absolution deviation of predicted E*_∞_* from experimental values are 0.08 and 0.11 eV, respectively, suggesting this approach could provide sufficient accuracy to be useful in polymer screening.

## Conclusion

The conceptual approach to low bandgap copolymers adopted in the present work allowed us to analyze and interpret both the benzo-quinoid and DA strategies to low-bandgap polymer design within the unified framework of the simple, but physically meaningful Kuhn model. The two parameters which determine the optical bandgap at the polymer limit (but also for oligomers) are essentially the bandgap of the repetition unit and the coupling between them. The latter is intimately related to the change of the BLA upon electronic excitation, which should be minimized to achieve low bandgaps and is favored by quinoidal structures in the ground state. In fact, the annulated electron-poor acceptor units generally used to generate low bandgap copolymers additionally enhance the quinoidal character in the polymer chain through resonance stabilization of the annulated rings. Because this can significantly vary depending on the D/A pairing, simple monomer-polymer correlation can be misleading, and the reliable performance of quantum-chemical methods on the chainlength evolution has to be carefully checked through proper comparison with experiments on (co-)oligomers. Such tests are also recommended before using the QC methods in periodic schemes.

Our tests on *n*T homo-oligomers and low bandgap *n*TPP co-oligomers included different TD-DFT methods ranging from the popular B3LYP and other hybrids incorporating higher fractions of HF exchange (BHLYP, M06HF) to LC functionals (CAM-B3LYP, and optimally gap-tuned OT-LC-BLYP). All methods with low to medium HF exchange including the LC variants significantly overestimated the slopes of the chainlength evolution and only occasionally predict polymer optical gaps in agreement with experiment in cases of fortuitous cancellation of errors, and hence cannot be recommended for the prediction of polymer bandgaps. TD-M06HF, when combined with planar M06HF geometries, results in slopes in very good agreement with experiment, except for systems which are clearly non-planar (e.g., *n*TDPP), for which it overestimates slopes. Removing symmetry constraints and re-optimizing non-planar geometries fixes the slopes of such non-planar systems, but overestimates inter-ring torsion angles in *n*Ts, resulting in underestimated slopes and a large blue-shift. The combination of non-planar BHLYP geometries and M06HF bandgaps seems to provide a more balanced description of both planar and non-planar systems, but with a slight loss of accuracy for both classes. For both approaches a strong hypsochromic offset is found, which however is fairly constant for the four series of in total 17 oligomers we have tested them on. For TD-M06HF/M06HF on planar geometries the offset is 0.75 eV with σ = 0.05, for TD-M06HF/BHLYP it is −0.70 eV with σ = 0.07. The largest error in *E_∞_* relative to extrapolated experiment values are 0.11 and 0.09 eV, respectively, suggesting that both approaches could be useful for predicting polymer bandgaps prior to synthesis. Nevertheless, it is expected that the ongoing development of new functionals will lead to further improvements in the description of the chain-length evolution of optical gaps of conjugated oligomers with non-negligible inter-ring torsion angles.

### Conflict of interest statement

The authors declare that the research was conducted in the absence of any commercial or financial relationships that could be construed as a potential conflict of interest.
